# Extracellular vesicles in osteoarthritis of peripheral joint and temporomandibular joint

**DOI:** 10.3389/fendo.2023.1158744

**Published:** 2023-03-06

**Authors:** Benyi Yang, Xin Li, Chaoran Fu, Wenyi Cai, Bowen Meng, Yan Qu, Xiaoxing Kou, Qingbin Zhang

**Affiliations:** ^1^ Guangdong Provincial Key Laboratory of Stomatology Hospital of Stomatology, Guanghua School of Stomatology, Sun Yat-sen University, South China Center of Craniofacial Stem Cell Research, Guangzhou, China; ^2^ Department of Temporomandibular Joint, Affiliated Stomatology Hospital of Guangzhou Medical University, Guangzhou Medical University, Guangzhou, China; ^3^ Guangzhou Key Laboratory of Basic and Applied Research of Oral Regenerative Medicine, Guangdong Engineering Research Center of Oral Restoration and Reconstruction, Guangzhou, China

**Keywords:** osteoarthritis, TMJ, peripheral joint, extracellular vesicles, EV therapy

## Abstract

Osteoarthritis (OA) is a disabling disease with significant morbidity worldwide. OA attacks the large synovial joint, including the peripheral joints and temporomandibular joint (TMJ). As a representative of peripheral joint OA, knee OA shares similar symptoms with TMJ OA. However, these two joints also display differences based on their distinct development, anatomy, and physiology. Extracellular vesicles (EVs) are phospholipid bilayer nanoparticles, including exosomes, microvesicles, and apoptotic bodies. EVs contain proteins, lipids, DNA, micro-RNA, and mRNA that regulate tissue homeostasis and cell-to-cell communication, which play an essential role in the progression and treatment of OA. They are likely to partake in mechanical response, extracellular matrix degradation, and inflammatory regulation during OA. More evidence has shown that synovial fluid and synovium-derived EVs may serve as OA biomarkers. More importantly, mesenchymal stem cell-derived EV shows a therapeutic effect on OA. However, the different function of EVs in these two joints is largely unknown based on their distinct biological characteristic. Here, we reviewed the effects of EVs in OA progression and compared the difference between the knee joint and TMJ, and summarized their potential therapeutic role in the treatment of OA.

## 1 Introduction

Extracellular vesicles (EVs) are nanoscale sphere-like phospholipid bilayer particles secreted by cells in a physiological or pathological state (1, 2). EVs can inherit bioactive substances from their host cells, including proteins, lipids, DNA, micro-RNA (miRNA), and mRNA (3). Nevertheless, EVs are not only carriers of bioactive substances. They also deliver their contents to target cells by specific ligand-receptor binding patterns or endocytosis (4). From this, EVs can mediate the communication between cells and affect the biological behavior of target cells (5). The diameter of EVs is generally 30-2000 nm. Based on particle size and generation pattern, EVs can be divided into three main types: exosomes, microvesicles, and apoptotic vesicles (apoVs) (6, 7). Exosomes exist in daily cellular activities and are usually considered to be intermediate between 30-150 nm. With the stage of endocytosis, sorting endosomes and multivesicular bodies, exosomes are finally assembled and released to specific tissue (8, 9). The diameter of microvesicles is larger than that of exosomes, which is about 200-1000 nm. Compared to exosomes formed by endocytosis, microvesicles shed directly from the cytoplasmic membrane (10). ApoVs are produced during the process of cell apoptosis, which include apoptotic bodies, apoptotic microvesicles, and apoptotic exosomes (11, 12). Different from other vesicles, the diameter of apoVs is variable and previous studies mainly focused on apoptotic bodies in 1-5 µm diameter (13–15). Recent studies show that apoptosis also encompass apoptotic microvesicles (100-1000 nm in diameter) and apoptotic exosomes (<150 nm in diameter) (16, 17). Emerging evidence shows EVs widely participate in cell activity and pathological processes.

As the population ages globally, joint trauma rate and the incidence of osteoarthritis (OA) are increasing yearly, affecting more than 240 million people and imposing a significant medical burden on society (18, 19). OA is a degenerative joint disease characterized by synovial inflammation, progressive cartilage degradation, and subchondral bone remodeling, leading to joint pain, deformity, and dysfunction (20). OA can affect both peripheral joints and temporomandibular joints (TMJ), which are synovial joints (21). The most affected peripheral joints are the joints of the fingers, knee, and hip joints. Their symptoms can interfere with work and normal daily activities (22). TMJ are the two synovial joints connecting the jawbone to the skull. Similar symptoms with peripheral and TMJ OA cause joint pain and movement limitation. Different from peripheral joint OA, TMJ OA is the terminal stage of TMJ disorder and often presents with abnormal jaw movement and restricted mouth opening, partly accompanied by tinnitus, headache, and other symptoms (23).

Traditional treatment emphasizes symptomatic treatment. However, it showed a limited effect in reversing the destruction of cartilage or subchondral bone (24). Although emerging stem cell therapies can promote cartilage tissue regeneration, shortcomings such as immune exclusion and tumorigenicity alarmed us (13, 25). The therapeutic effects of stem cells are mainly attributed to their paracrine effects, and EVs are one of the important components of paracrine secretion (26). Compared with stem cell therapy, EVs have many superiorities, including explicit pathways of effect, low immunogenicity, low tumorigenicity, easy preservation, and no need to consider cell survival and abnormal differentiation (27). So EVs possess a safer and greater tissue regeneration characteristic. Mesenchymal stem cell (MSC)-derived EVs have been shown to have notable effectiveness in OA on pain relief, inhibition of inflammation, immunomodulation, and cartilage tissue regeneration (28–31).

Furthermore, EVs carrying specific substances can be used as potential biomarkers for the diagnosis of OA and for monitoring the progression of OA (32). EVs, as a future cell-free therapy, show promising applications in joint diseases. It is expected to become a better alternative to MSC therapy in tissue regeneration. Although the significance of EVs in the pathogenesis and treatment of OA has been reviewed elsewhere (33–36), there is still lacking a review that summarizes the similarities and differences about EV function between peripheral joint and TMJ OA. This manuscript mainly compares the difference between the peripheral joints and TMJ and reviews the role of EVs in the pathogenesis and treatment of peripheral joints OA and TMJ OA. We also highlight the challenges facing EVs used as a conventional biologic agent for the treatment of OA and the significance of exploring next-generation EV-drug in the future.

## 2 The difference between peripheral joint and TMJ

### 2.1 The anatomy and development in TMJ and peripheral joint

The difference between the peripheral joint and TMJ is mainly generalized for anatomy and development. Peripheral joints include knee, hip, shoulder, elbow, wrist, and ankle joints, most of which are subordinate to synovial joints. The typical structure of synovial is comprised of two opposing skeletal elements and intermediate discs capsuled by synovial tissue, permitting a wide and low-friction movement (37). The knee is the largest synovial joint and is studied the most. Here, we used the knee joint as a representative to compare with TMJ, which both belong to synovial joints. As a typical single synovial joint, the knee comprises the patella, femur, tibia, fibula, meniscus, and surrounding ligaments and joint capsule (38). The TMJ also comprises the synovial joint capsule and ligaments, temporal fossa, articular tuberosity, mandibular condyle, and articular disc (39). Besides the typical synovial joint structures, TMJ is the only bilaterally linked joint characterized by sophisticated structure, precise regulation, and complicated functions (40). The other anatomical difference is the nerve distribution, which may be the basis for the distinct symptoms of TMJ OA. No sensory structures are included within the 3 cm diameter sphere centered on the meniscus of the knee. In contrast, several important anatomical structures and sensory nerves are within the 3 cm diameter sphere area centered on the TMJ disc, such as the cochlea, brain, trigeminal ganglion, mandibular nerve, and auriculotemporal nerve (41). The nerve-rich structure may be the reason that TMJ OA patients can be more susceptible to neurological symptoms (42).

**Figure 1:**
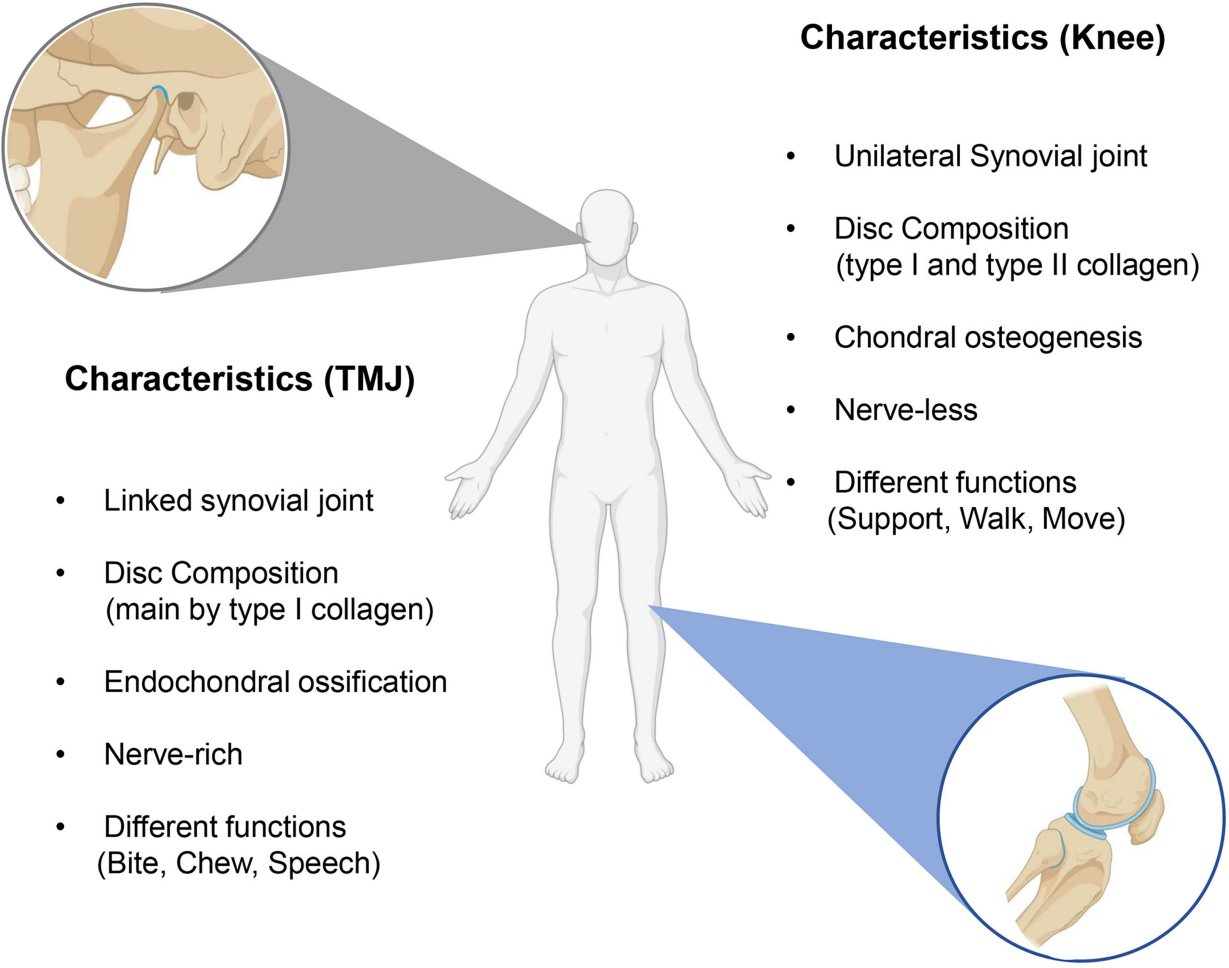
The differences between knee OA and TMJ OA. The main differences are the histological structure and functions.

The different growth pattern is the other reason for TMJ specificity. TMJ is derived from the cranial neural crest in the way of intramembranous ossification, whereas peripheral joints are mainly derived from cell migration of lateral plate mesoderm by endochondral ossification, which may contribute to the histological differences between the two joints (43). The articular cartilage type on the surface of the condyle and the knee joints is distinguishable. It is known that the articular surface of the knee is covered by hyaline cartilage with varying proportions of type I and type II collagen, whereas the surface of TMJ is covered by fibrocartilage and is composed mainly of type I collagen (32). In addition, the growth pattern of condylar is different from the long bones. Condylar have ever been regarded as a semi-epiphyseal plate of long bone, but now it has been proven to be wrong (44). The condylar cartilage derived from the cranial neural crest is secondary cartilage, undergoes endochondral ossification, and exhibits characteristic developmental processes, whereas the cartilage of long bones directly originates from embryonic cartilage primordia (45, 46) (**Figure 1**).

### 2.2 Clinic manifestation in TMJ OA and peripheral joint-OA

OA is a common chronic degenerative joint disease worldwide, which is believed to be the fourth top cause of disability worldwide (22). OA is usually associated with genetics, trauma, old age, obesity, mechanical stress, mental elements, and other factors. However, the pathogenesis of OA is still unclear (47, 48). Typical clinical symptoms of knee OA are often joint pain and limitation of movement. Patients suffering from knee OA usually feel pain and transient morning stiffness at the early stage. With the progress of OA, a more severe lesion can be found, including subchondral bone cysts, bone marrow lesions, and osteophytes. These changes can be diagnosed by Cone Beam Computed Tomography (CBCT) and Magnetic Resonance Image (MRI) with osteophytes, bone marrow lesions, and meniscal tears (49). Despite similar joint symptoms, mental and biopsychosocial symptoms are also crucial in TMJ OA. TMJ OA presents hyperalgesia and neurological symptoms, including tinnitus, headache, and psychological disabilities (50). In addition, CBCT is relatively mild and often shows blurred and incomplete condylar cortex, localized bone resorption defects, bone redundancy formation, subchondral sclerosis, or cystic bone changes (51). The current goals for treating knee and TMJ OA are consistent, include: reducing pain in the joint area, ameliorating joint function, and slowing the progression of OA. Most patients are treated clinically by a combination of conservative and surgical treatment, which requires a personalized treatment plan that considers the patient’s situation, such as age and severity of the disease (52). In addition to traditional treatments, such as physical therapy, oral medications, intra-articular injections, minimally invasive arthroscopic surgery, and surgical operation, many emerging biological therapeutic approaches are currently under study, such as stem cell therapy, platelet-rich plasma, stromal vascular fraction, and EVs (53–56).

### 2.3 Animal models of OA and TMJ OA

**Figure 2:**
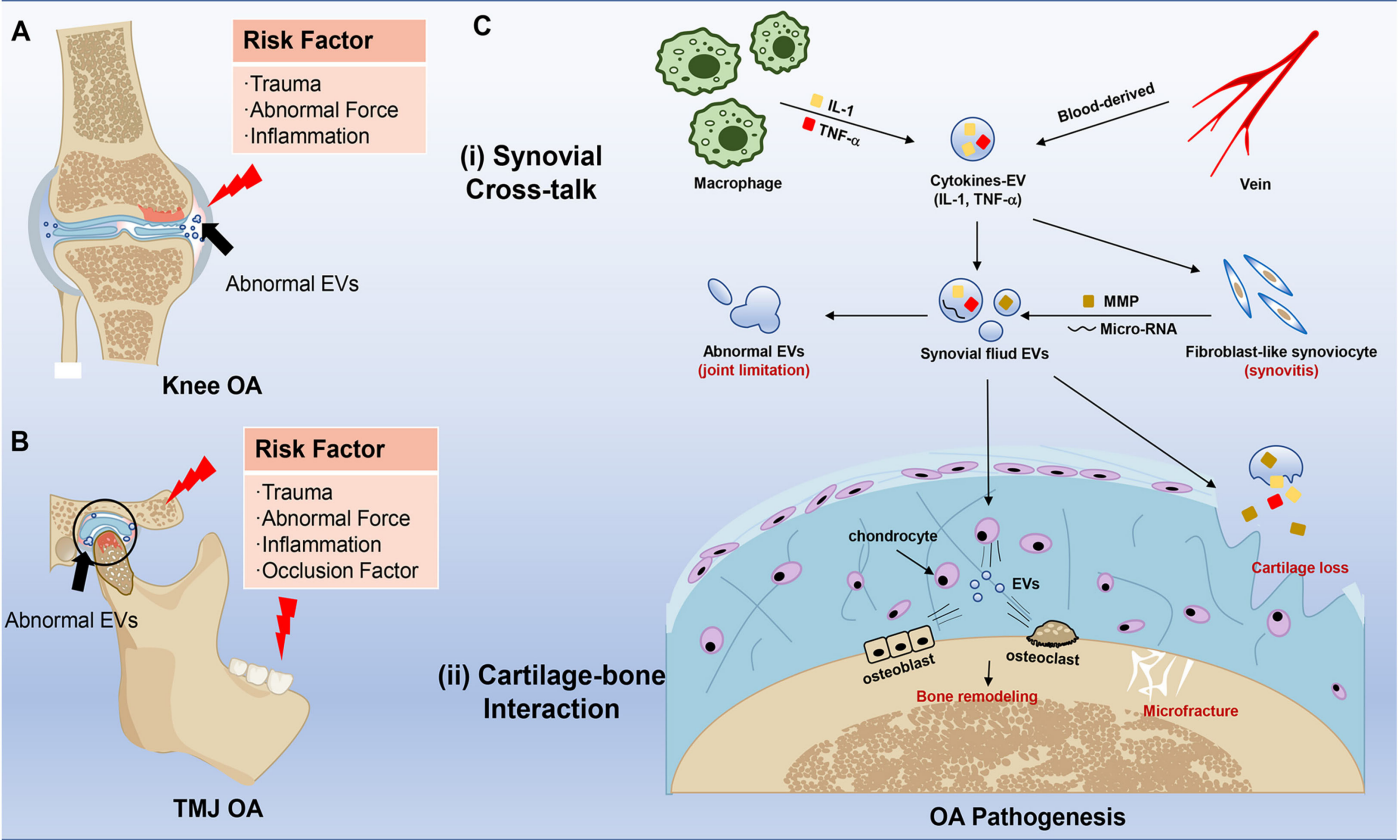
Risk factors and effects of EVs in knee OA and TMJ OA pathogenesis. **(A)** Risk factors in knee OA. The red pattern means inflammation and bone destruction, and the blue pattern means healthy joint with uniform EVs. The black arrow points to abnormal EVs in the joint. **(B)** Risk factors in TMJ OA. The red pattern means inflammation, and the black arrow points to abnormal EVs. **(C)** Schematic representation of EVs in the pathogenesis of OA. The red typeface stands for clinical change in OA. The major effects of EVs are mainly divided into two sites, including (i) Synovial Cross-talk and (ii) Cartilage-bone Interaction. The two sites also communicate with EVs.

OA is a multifactorial disease, mainly including aging, obesity, anatomical factors, mechanical loading, and genetic factors (**Figure 2A**). Despite all the above elements contributing to TMJ OA. Occlusion elements, mental and biopsychosocial factors also play an important role in the pathogenesis of TMJ OA (22, 50) (**Figure 2B**). Based on these risk factors, OA animal models are usually divided into induced, naturally occurring, and genetically modified models, while the knee is the most used joint for the animal OA model. Similar invasive approaches are suitable for both knee and TMJ, including surgical induction and chemical injection (monosodium iodoacetate, papain, collagenase). In comparison, the surgical methods to induce knee and TMJ OA are distinguished based on different anatomical structures (57). The differences between knee and TMJ OA models mainly display in non-invasive methods.

Although the high-fat diet model and mechanical loading can apply to both knee and TMJ, more specific methods are required for TMJ OA (58, 59), including but not limited to disordered bite, excessive mouth opening, and soft food induction (58, 60). Intriguingly, the sleep deprivation model can be used to reduplicate the model of TMJ OA, which further explains the relationship between mental factors and TMJ OA (61). In addition, naturally occurring and genetically modified models are also used to study the progression of OA to simulate aging and genetic factors, which have been reviewed in detail (57). OA is a multi-factor process, and most animal models are single-factor models. It reminds us that choosing the appropriate animal model needs strict consideration according to the purpose of the study.

## 3 The role of EV in the pathogenesis of OA and TMJ OA

The pathogenesis of OA is complicated and diversified. It is not only a single disease, but a common final stage of joint failure contacted with body status, environmental impact, and triggered by other risk factors (62–64). In current views, OA is a degenerative disease characterized by the destruction of cartilage, synovial inflammation, and bone remodeling increase (64–66). These changes can be observed in the early stage of OA (49). OA usually needs several years to develop, meaning this disease needs long-term pathological stimulus and progresses to a worsened state.

EVs distribute in humor and tissues, deliver bioactive substances, and participate in cellular activity, which plays a vital role in the whole progression of OA. On the one hand, the serum-derived EVs carried bioactive substances like IL-1β which activated fibroblast-like synoviocytes (FLS) (67), and then these EV cargos can further raise monocytes and macrophages, which usually barely exist in normal synovial tissue. Then a low-grade inflammation can be continuous in synovial fluid and tissue in the whole stage of OA (68). On the other hand, synovitis is closely related to cartilage destruction (69, 70). Activated FLS can secret more EVs carrying inflammatory factors, micro-RNA, and matrix metalloproteinases (MMPs) to induce the apoptosis of chondrocytes and destroy the extracellular matrix (ECM) (71–73) (**Figure 2C**). So, it is essential to understand EVs’ function in OA seriously.

### 3.1 Inflammation and cytokines

OA has been described as an inflammatory disease for a long time. Many individuals with OA have joint inflammation symptoms like stiffness and pain. In addition, low-grade inflammation can be observed in patients during the whole progression of OA (74). The previous study showed that OA patients have a high level of inflammatory plasma proteins such as albumin and acid glycoproteins in their blood and synovial fluid (75). Recent research showed that other inflammatory factors also maintain a high concentration in OA synovial fluid, including complement components and cytokines (IL-1β, TNF, IL-6, IL-8) (76–79). Furthermore, these inflammatory mediators can be produced or overproduced by chondrocytes and synovial cells in OA (78). These evidences showed a close relationship between inflammation and OA.

Undoubtedly, inflammation factors are crucial in OA progression. These cytokines possess little organizational penetration and stability, so EVs are needed for bioactive cytokines carry to penetrate into deep cells or tissues (34, 80, 81). As a cell secretory mediator, EV has been demonstrated to contain many cytokines, miRNA, and other bioactive substances. EVs can be endocytosed by chondrocytes, FLS, and macrophages which are the main cells in the joint cavity microenvironment (82–84). These biological characteristics are the basis of EV to be effective. In knee OA, a mass of EVs protein cargoes has been identified, including inflammatory cytokines (for example, IL-1, IL-6, and TNF-α), immunoglobulin, complement component, fibrinogen, apolipoprotein and transforming growth factor β (35). EVs derived from synovial fluid of severe knee OA contained higher levels of numerous cytokines (85). Among all the cargoes of EVs in OA, two major players, IL-1β and TNF-α, can induce cartilage destruction and inflammation (71). It was well known that IL-1β and TNF-α increased in synovial fluid, synovial tissue, and cartilage of OA patients (86). On the condition of OA, the plasma EVs can carry a mass of TNF-α to participate in OA progression (87). Similarly, the EVs in OA patients’ synovial fluid also carry many inflammatory cytokines, including IL-1β and TNF-α (88). Subsequently, FLS strongly expressed IL-6, IL-8, and MMP, which can directly destroy the extracellular matrix stimulated by activated or apoptotic T cells and macrophages EV (89). This process demonstrated that the EVs from arthritis patients’ synovial fluid could further induce the FLS, secreting inflammatory cytokines and chemokines (90). FLS and immunocyte-derived EVs formed a high-concentration EV microenvironment in synovial fluid. These EVs can further be endocytosed by chondrocytes, which trigger chondrocytes to produce more inflammatory cytokines and chemokines (33). The other experiments demonstrated that the healthy chondrocytes treated with OA-derived EVs displayed elevated expression of inflammatory genes (88). OA is the result of the cascade reaction of inflammation. Immunocytes, FLS, and chondrocytes can be activated by EVs with different components and finally induce the production of MMP and destruction of ECM. Overall, the progression of OA in different joints is similar. The changes in knee joint OA can also be detected in TMJ OA (91–94). More and more evidence prove the relation between OA histological changes and EVs. However, the mechanism between the biological changes of EVs and synovial inflammation and cartilage loss is still unknown.

### 3.2 MicroRNA

People have realized the effect of miRNA on OA progression in recent years. miRNAs are a family of approximately 21-nucleotide-long RNAs that can regulate genetic expression by combining the 3’-UTR (95, 96). MicroRNA gets involved in all the known cellular activity and widely participates in disease progression, including OA (95). It has been demonstrated that miRNA differential expression is the characteristic of OA and EV-microRNA is highly related to OA progression (35, 49, 88). Compared with healthy people, the EVs from synovial fluid of the OA knee joint showed a differential microRNA pattern (88, 97). Recent research showed that 142 microRNAs were differentially expressed between damaged or non-damaged articular cartilage in peripheral joint OA patients (hip and knee) (98). Moreover, researchers collected the human synovial fibroblasts from healthy knees to verify *in vitro*. Likewise, approximately 340 miRNAs upregulated in FLS treated by IL-1β; meanwhile, only 11 miRNA increased in FLS-derived EVs (99).

The synovial fluid-derived EV-miRNA can be endocytosed by chondrocytes and stimulate inflammation in cartilage (88). Since EV-miRNA penetrated into cells, they can directly bind to specific mRNA or proteins to regulate cellular behaviors. Present research mainly focused on knee OA. For example, miR-181a-5p is a critical mediator involved in the cartilage destruction by promoting inflammatory, catabolic, and cell death activity (100, 101). Notably, not all the OA patients have the same EV-miRNA changes; EV-miRNA in OA displays a gender-specific pattern (88). Only one miRNA (miR-504-3p) were upregulated in both genders, which may promote cell apoptosis (88, 102). The other downregulated miRNAs were associated with cell adhesion molecules, whereas the upregulated miRNAs were related to biotin metabolism signaling. The female-specific EV-miRNAs most participate in estrogen pathway (35), which may explain the high incidence rate of OA in female (22).

TMJ OA also has similar mechanisms that microRNA can anchor target pathways to regulate OA progression, including Wnt, Smad/TGF-β, and PTEN (103–106). Like knee OA, TMJ OA also displays a high incidence rate in females, which may attribute to EV-miRNAs gender-specific expression. However, TMJ OA is different from knee joint OA, such as gender incidence rate and symptoms, and there is little research to detect the difference in TMJ OA.

### 3.3 Mechanical stimulation

It is undisputed that the mechanical factor is the key to OA progression. The joint is the force-bearing structure of our bodies. Owing to the existence of synovial fluid and disc, bone can move with very low friction. Even so, the response of different joints to force-loading is usually discrepant. For example, the knee usually bears a relatively large force, four times more than body weight in jogging, while TMJ can undergo a body weight when people bite (107, 108). Different function determines different mechanical adaptation, and overuse may cause joint structural changes. So, articular inflammation mainly concentrates on the functional joints like the knee, hip, hand, and TMJ (49). With further validation, we gradually realize that the mechanical factors are not just the physical “erosion” from the previous studies (109). It is a complicated system composed of mechanically sensitive pathways and functional proteins. In the early stage of OA, the microfracture began to emerge, and the cartilage was the first to appear in the microarchitectural changes with chondrocyte mitochondrial dysfunction (110, 111). The mechanical loading may further impact the chondrocyte, which secreted EVs containing mechanically sensitive substances like miR-221-3p to regulate cell communication in bone remodeling (112). However, the impact of mechanical is usually self-limiting. If we intervene timely, the cartilage dysfunction will recover and rebalance.

In the process of force loading, overuse or abnormal loading can cause cartilage loss and subchondral bone marrow lesions with meniscus degeneration or severe tears. Unlike the knee joint, TMJ are two linkage joints having a more precise mechanical system. Recent studies showed that increased loading force led to a thicker calcified cartilaginous layer of TMJ and caused osteochondral interface stiffness with mild disc displacement or perforation (110, 113). Although there is a difference in symptoms between the two joints, the initial mechanical response systems are both chondrocytes and ECM. EVs are one of the most important components in ECM and mediate cell-to-cell interaction in this system (114). In fact, it can detect a mass of apoVs in the ECM collected from knee OA (115, 116). ApoVs were also observed in the apoptotic chondrocytes of TMJ OA (117). Except for apoVs, the other EVs are mainly from articular cartilage that contains over 1700 bioactive substances, including type II transglutaminase, COL II, aggrecan, βig-H3 (TGFβ-induced protein), and GAPDH (80, 118). These EVs form the ECM and regulate the communication between cartilage and bones (119). In the process of knee OA, as the force loading, ECM-derived EVs respond to mechanical effects and are released by chondrocytes, and then interact with the surrounding cells by activating bone morphogenetic proteins (BMPs) and transforming growth factor-β (TGFβ). These changes will further activate downstream pathways, including Wnt and MAPK signaling pathways (109). Finally, these ECM-derived EVs can take away the ECM components, which contains lots of cytokines and cathepsin, such as MMP-2, causing cartilage loss and bone lesions (120). In addition, not only the cargos but EVs’ mechanical properties also impact the progression of OA. Studies showed that EVs in OA become inhomogeneous and soft (121), which indicated that mechanical loading may change the mechanical properties of EVs derived from cartilage and bone.

The TMJ OA showed a similar pathological process with knee OA by abnormal mechanical stress stimulation. These EVs and changes can also be found in TMJ OA, including TGFβ, BMPs, Wnt, and FGF pathways (60, 122). However, there are still some differences between TMJ OA and knee OA. Considering they have different origins and growth patterns, the expressions of the same genes also cause different endings. For example, small leucine-rich proteoglycans (biglycan/fibromodulin) are one of the ECM components, participating in both knee OA and TMJ OA (123). Intriguingly, TMJ OA was observed four months later than knee OA in biglycan/fibromodulin double-deficient mice (124, 125). Although we have reviewed current research, the specific pathways of knee and TMJ OA are still unclear. Overall, more direct evidence should be presented in mechanical-induced OA.

### 3.4 Genetic factor

OA is regarded as an idiopathic disease; however, genetic factor is also an important reason for OA development. OA shows the characteristic of familial aggregation, and the heritability rate of OA is around 35%-65% depending on different joints (126, 127). OA is a polygenic disease that cannot attribute to any single gene. Gene detection was done to analyze the susceptible gene including growth differentiation factor 5 (GDF5) and human leukocyte antigen (HLA) class II/III locus (126, 128). A European genome-wide association study (GWAS) confirmed two genes were related to knee pain in 171561 people, which were close to GDF5 and COL27A1 (129).

Compared with the early industrial and prehistoric eras, the OA prevalence rate of modern people has doubled (130). These genes have so low odds ratios that they are less responsible for OA. However, the paradoxical evidences seem unreasonable to explain the familial aggregation and mother-daughter inheritance patterns in OA. Recent research showed that mothers suffering from OA were more likely to pass it on to their offspring compared with fathers (131). Mother-to-child transmission patterns contain mother-to-embryo, which mainly depends on EVs except for gene recombination. Since mothers are inflammation sufferers, EVs can carry these inflammatory cytokines (IL-1β, TNF-α) and environmental factors, pass through the placental barrier into the fetus (132). These studies added EV regulatory factors to explain why OA presents as a matrilinear inheritance with a low-degree effect of susceptible genes. However, further studies are needed to elaborate.

## 4 The role of EVs in the treatment of peripheral OA and TMJ OA

The treatment of OA is always challenging. Currently, conservative and surgical treatments are used to treat OA, but there is no golden therapy for OA. The mild OA lesions can be treated with drugs or splints, while the severe ones may need surgical therapies. In 1957, the first hemiarthroplasty knee device was designed by McKeever, while Christensen designed a fossa-eminence prosthesis for TMJ hemiarthroplasty six years later (133, 134). Till now, knee arthroplasty and hip arthroplasty have been well developed, but TMJ arthroplasty rarely achieves satisfactory results (135, 136). With the progression of OA research, people have gradually realized the importance of biological factors in OA treatments. In 1985, IL-1 receptor antagonist (IL-1Ra) was first reported to find in human macrophages and synovial macrophages in OA patients (137). Autologous conditioned serum (ACS) therapy, mainly working through IL-1Ra, was studied in the mid-1990s (138). The preparation of autoserum is a mature technological process, and the autologous platelets can produce IL-1Ra and various kinds of cytokines in cell degranulation (139). People realize that IL-1Ra was not the only one in OA, and platelet-rich plasma (PRP) was studied subsequently (140).

Because of their preparation process, ACS and PRP retain lots of blood-derived EVs, which showed therapeutic effects in OA (141, 142). It was reported that blood-derived EVs partook the substance exchange and cell communication (141). Blood-derived EVs can inhibit inflammation and elicit chondroprotective gene expression (142). In addition, a recent study showed that blood-derived CD34^+^ EVs were the potential therapeutic targets for OA. Blood-derived CD34^+^ EVs were the only subpopulation that significantly correlated between plasma and synovial fluids containing lots of functional mitochondria and little pathogenic cytokines such as TNF-α and IFN-γ (143, 144). EVs from autologous blood-derived products may be the core components of treating OA. Although ACS and PRP showed significant effects in animal models, the complexity of autologous blood-derived products caused controversial clinical efficacy (145, 146). More biotherapies have been studied, including MSC therapy and EV therapy (34, 147). Compared with traditional stem cell therapy, EV administration does not have the risks of allogeneic and xenogeneic immunological rejection and malignant transformation, so it has opened a new era of stem cell therapy.

### 4.1 Stem cell therapy for OA

With the development of regenerative medicine, stem cell therapy was gradually studied. OA is a degenerative disease, so researchers mainly focused on tissue regeneration and immunoregulation, and stem cell meets requirements. MSCs have the characteristics of self-renew, multiple differentiation, and immune regulation, which contribute to tissue repair of OA (148–151). The clinical trial also showed that stem cell therapy could effectively treat knee OA (152). A recent multicenter randomized controlled clinical trial (phase I/II) demonstrated that bone marrow MSC (BMMSC) therapy could safely alleviate the symptoms of knee OA (153). In contrast, the successive clinical trial showed no significant difference between PRP therapy (154). In the treatment of TMJ OA, stem cells from different sources showed therapeutic effects and promoted condylar cartilage regeneration (24, 155–158). In addition, up to 2,021 randomized clinical trials of stem cell therapy showed a statistically significant superiority over hyaluronan in pain relief and mandible mobility (159). However, stem cell therapy has disadvantages, including immunogenicity and unstable phenotype, so it reminds us whether there is an alternative approach in biotherapy which can be more effective for OA (27).

### 4.2 EV treatment for peripheral joint OA

Compared to cells, EVs show lower immunogenicity. Therefore, it is safer to apply EVs in OA treatment than cells (160). EVs from a variety of sources, including synovial MSCs (SMSCs) (161–163), adipose-derived MSCs (ADMSCs) (164, 165), BMMSC (166), and human umbilical cord MSCs (hUMSCs) (167, 168), has been proved to be beneficial for peripheral joint OA treatment.

Perturbed balance of the local immune system is the key factor that causes clinical symptoms and organic damages in OA. During knee OA development, immunological cells exert proinflammatory factors, such as IL-1, IL-6, IL-8, and MMP-3, thereby inducing synovial inflammation and cartilage degradation (169). MSC-EVs have immunomodulatory effects in peripheral joint OA treatment. MSCs-EVs generate large amounts of anti-inflammatory cytokines, such as IL-10 and TGF-β1, and simultaneously suppress the production of the proinflammatory factors IL-1, IL-6, TNF-α, and IL-12. Moreover, MSC-EVs inhibit macrophage activation and induce the proinflammatory M1 phenotype to convert to the anti-inflammatory M2 phenotype. Together, MSC-EVs decrease local inflammatory reactions in knee OA (170).

Inflammatory response causes cartilage degeneration in peripheral joint OA, including cell death, matrix degradation, and finally, a loss of structure and function (160, 171). MSC-EVs prevent chondrocyte apoptosis (170) and facilitate cell migration and proliferation in peripheral joint OA (172). By mediating the expression of fibroblast growth factor (FGF)-2, survivin, and Bcl2/Bax, EVs directly promote chondrocyte proliferation or eliminate the inhibitory effect of pro-inflammatory factors like TNF-α and IL-1β (167, 170, 173, 174). Besides, MSCs-EVs can also control chondrocyte proliferation and migration by releasing miRNAs (164, 175, 176).

**Figure 3:**
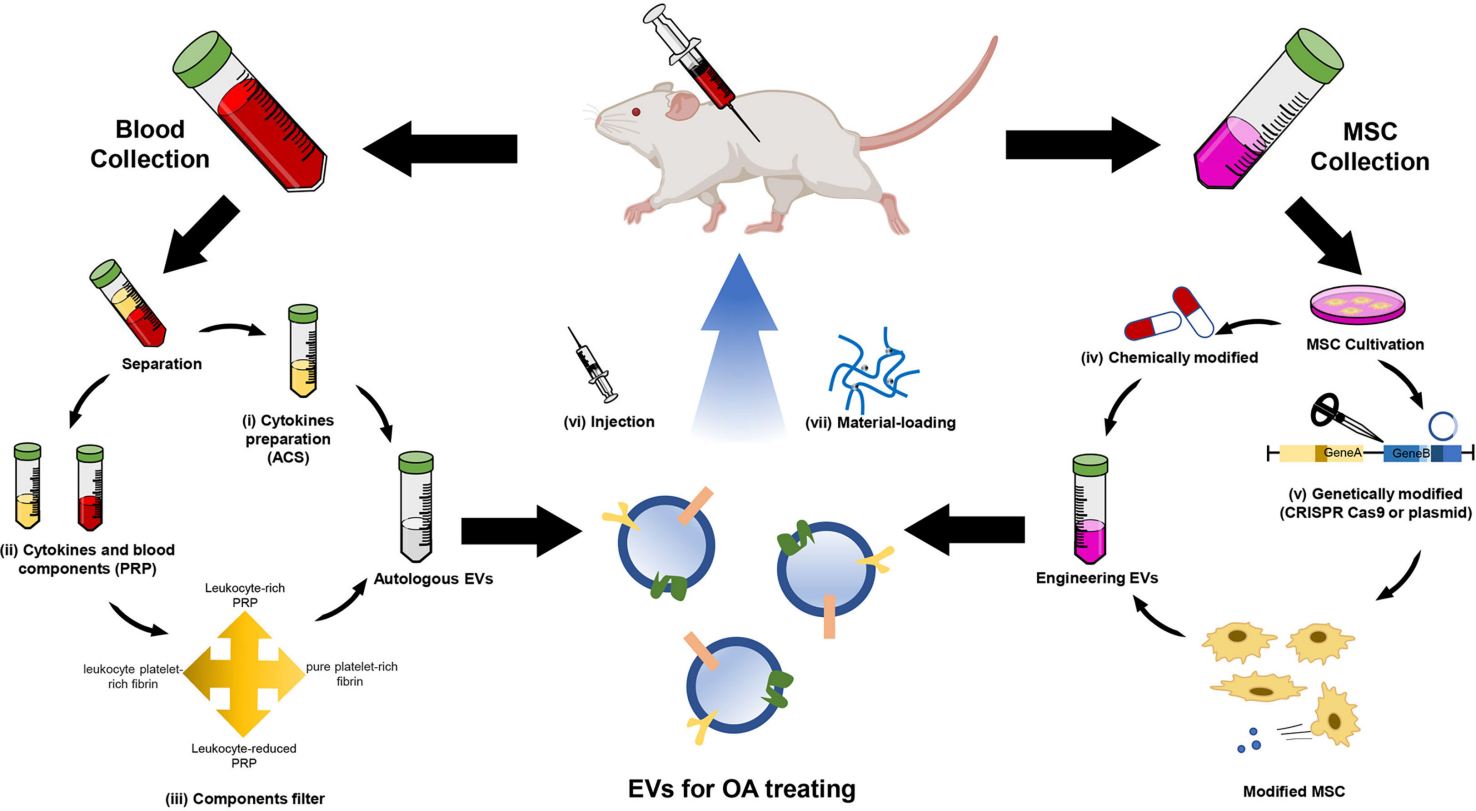
Schematic illustration of EV therapies for OA. EV therapy usually collects EVs from autologous EVs and engineering EVs. The autologous EVs are mainly derived from (i) Autologous conditioned serum (ACS) and (ii) platelet-rich plasma (PRP). The latter needs (iii) components filter and then produces four types of EV suspension. The engineering EVs are mainly derived from MSCs. Two strategies are used to modify the MSC-derived EVs, including (iv) chemical and (v) genetic modification. The modified MSC-secreted specific EVs contain miRNA and proteins in OA treatment. Autologous or engineering EVs are usually delivered by (vi) injection and (vii) material-loading.

MSC-EVs induce the expression of matrix protein, while inhibit the expression of matrix-degrading enzymes, thereby promote ECM synthesis. MSC-EVs can reduce the expression of MMP-1, MMP-3, MMP-13, and ADAMTS-5 and increase COL II production (165). Previous research has elucidated that MSC-EVs regulate the expression of cartilage formation-related genes such as aggrecan, SRY box gene-9 (SOX9), COL9A1, COL2A1, and cartilage oligomeric matrix protein (COMP) while decreasing the expression of COL10A1, Runt-related transcription factor 2 (Runx2), and MMP-13, with their cargos, including miRNAs and proteins (177). Besides the natural EVs, there are emerging types of engineered EVs. Engineered EVs are designed for targeted therapy or controlled release and have better therapeutic properties, which have drawn increasing attention of researchers (34, 178, 179) (**Figure 3**).

### 4.3 EV treatment for TMJ OA

Studies on TMJ OA are relatively stagnant compared with knee OA (41). To date, few studies have demonstrated the effect of EVs on TMJ OA treatment. Based on current understanding, the mechanisms of EV therapeutic effect on TMJ OA are rather similar to OA. BMMSC-derived EVs could induce cartilage reconstruction in TMJ OA *via* the autotaxin–YAP signaling axis in chondrocytes (180). MSC-EVs activate AKT and ERK signaling in chondrocytes and then enhance the proliferation and migration of chondrocytes. The EVs can also activate AMP-activated protein kinase (AMPK) signaling, which contributes to restoring and maintaining chondrocyte matrix homeostasis (181). Moreover, MSC-EVs promote s-GAG synthesis and COL II protein expression, and suppress NO and MMP-13 production, to restore stromal homeostasis in a TMJ-OA, enhancing cartilage regeneration in chondrocytes (182).

### 4.4 Limitations of EV therapy in OA

EV therapy in OA still has limitations, including but not limited to the unclear mechanism, targeting and stability, and EV production. Although the engineered EVs exhibit targetable and stable properties, it is still unclear whether they may cause other problems (183). Moreover, there are difficulties in the preparation and preservation of EVs. The contamination from the EV separation process reduces the purity of EVs, and these confounding factors hamper EV usage in clinical practice (184, 185). Solving these problems is challenging, and more efforts should be devoted to EV research.

## 5 Conclusion and perspective

In summary, researchers have gradually realized the crucial function of EVs in OA progression. The effect of EV in OA is multiplex. EV-carried bioactive substances mediate cell-to-cell communication, and so does inflammation. Inflammation is passed and aggravated by EVs. Differed from Rheumatoid arthritis, which has a heavier inflammatory burden stimulated by immunocomplex than OA. EVs in OA cannot cause much stronger inflammatory storms, which may be attributed to their relatively low immunogenicity. The changes in EVs’ mechanical properties may limit joint movement. Meanwhile, the EV-carried bioactive substance may determine the fate of FLS and chondrocytes. This evidence suggests that OA is not only an inflammatory disease but also an EV-related disease.

There is no specific therapy for OA so reducing the risk factor is crucial to OA treatments. Although the joint replacement therapy succeeded in knee and hip, it was mainly for later period OA patients, and the treatment in TMJ failed. EV therapies may be a promising treatment for OA in peripheral joints and TMJ because of their low immunogenicity, low cytotoxicity, and long-term stability characteristics. Recently, the EVs from modified cells or loaded with drugs showed therapeutic effects in OA (182, 186, 187), which are attractive for this field. There are three forms of EVs, and the recent research of EV therapy mainly focused on exosomes, which showed therapeutic effects in OA treatment (188). Compared with exosomes, apoVs can be easier to obtain from tissues. It also contains more bioactive substances suitable for immunomodulation and regeneration (189, 190). Although there is currently no study in the treatment of OA with apoVs, considering its biological characteristics, we believe apoVs may be a potential candidate for OA therapy. However, the side effects of EVs are still unclear, which impels us to conduct a long-term safety assessment before the clinical transformation.

## Author contributions

BY and XL wrote the initial draft. CF, WC, BM and YQ contributed to the manuscript revision and figure preparation. XK and QZ provided their conception, design and comments on the manuscript and approved the final version of the manuscript. All authors contributed to the article and approved the submitted version.
